# What We Want to Know

**Published:** 1896-06

**Authors:** 


### WHAT WE WANT TO KNOW.

Why German horsedealers and others are placing every obstacle in the way of the introduction of American horses throughout their country, ten thousand of which were used by the German people last year? We have here a type of draught-horse better than they can buy in any other market, more suitable to their work and manner used than any others, and we can sell them at better prices. A mere prejudice will not do.

How four prominent veterinarians failed in an examination for soundness to note the existence of scirrhous cord in the trotting-bred horse "Sheraldine," recently sold at auction in Chicago?

Why the special report on diseases of the horse, issued by the Department of Agriculture during the term of Secretary Rusk, is now being hawked about by the second-hand bookstores of the country at the price of one dollar per copy?

Messrs. Parke Davis & Co. have kindly favored us with one of their new catalogues, containing a complete list of their large and varied number of preparations. It should be in the hands of every veterinarian, for it is an epitome of valuable information for every-day use and acquaints one with all the ideal plans and methods of preparation of the various drugs and chemicals in use for the relief and cure of disease.

